# Number Concepts without Number Lines in an Indigenous Group of Papua New Guinea

**DOI:** 10.1371/journal.pone.0035662

**Published:** 2012-04-25

**Authors:** Rafael Núñez, Kensy Cooperrider, Jürg Wassmann

**Affiliations:** 1 Department of Cognitive Science, University of California San Diego, La Jolla, California, United States of America; 2 Institut für Ethnologie, Universität Heidelberg, Heidelberg, Germany; Indiana University, United States of America

## Abstract

**Background:**

The generic concept of number line, which maps numbers to unidimensional space, is a fundamental concept in mathematics, but its cognitive origins are uncertain. Two defining criteria of the number line are that (i) there is a mapping of each individual number (or numerosity) under consideration onto a specific location on the line, and (ii) that the mapping defines a unidimensional space representing numbers with a metric — a distance function. It has been proposed that the number line is based on a spontaneous universal human intuition, rooted directly in brain evolution, that maps number magnitude to linear space with a metric. To date, no culture lacking this intuition has been documented.

**Methodology/Principal Findings:**

By means of a number line task, we investigated the universality proposal with the Yupno of Papua New Guinea. Unschooled adults did exhibit a number-to-space mapping (criterion i) but, strikingly, despite having precise cardinal number concepts, they located numbers only on the endpoints, thus failing to use the extent of the line. The produced mapping was bi-categorical and metric-free, in violation of criterion ii. In contrast, Yupnos with scholastic experience used the extent of the segment according to known standards, but they did so not as evenly as western controls, exhibiting a bias towards the endpoints.

**Conclusions/Significance:**

Results suggest that cardinal number concepts can exist independently from number line representations. They also suggest that the number line mapping, although ubiquitous in the modern world, is not universally spontaneous, but rather seems to be learned through — and continually reinforced by — specific cultural practices.

## Introduction

Mathematics is a fundamentally abstract human conceptual system. The degree to which mathematics is grounded in universal, biologically endowed intuitions, is still an open question [1], [2], [3], [4], [5]. One such intuition, the generic number line — simple, yet fundamental to mathematics — has been argued to be universal and rooted in brain evolution, emerging spontaneously early in ontogeny independently of education and culture [6]. Abundant research points to space as a natural source for number intuitions. Already in 1880 Francis Galton described, via introspection, that for some people numbers are pictured on a line [7]. Anthropological studies have documented that some groups with little or no exposure to formal schooling in remote areas map counting numbers to space by assigning them to a body part sequence [8]. More recently, developmental studies have shown that 5-year old pre-school children [9] and even 8-month old infants [10] appear to exhibit numerosity-space associations (but see [11] for a different interpretation). Studies in neuroscience and psychology on Western adults have shown that unidimensional space, supported by space-related brain areas [12], [13], [14], [15], plays a major role in number processing [16], [17], leading to the claim that numbers are represented in the human brain in the form of a mental number line [18], [15], oriented left-to-right and localized bilaterally in the intraparietal sulcus [14]. Furthermore, number estimation research has shown that number lines are highly intuitive, and one of the key methodologies in the field makes explicit use of an external number line [19], [20]. This number line task, where participants are asked to locate numbers on a line marked with a beginning number (usually 0 or 1) on the left endpoint and a larger number (often 100 or 1000) on the right endpoint, has been reported to be readily understood even by kindergarteners [19], [20] and unschooled people from indigenous cultures [6], and has thus been assumed to provide straightforward behavioral evidence of number line intuitions.

In this report we investigate the generic concept of *number line* as used in the fields of number cognition, child psychology, and cross-cultural studies, which primarily consists of a mapping of numbers (especially whole and natural numbers) onto a straight line. We therefore do not necessarily assume a technical connotation that the line is a graphical depiction of the set of real numbers. Such a concept, technically called *real number line* or *real line*, is more sophisticated, building on the concept of *complete ordered field* as specified by the *least upper bound axiom* among others, and it is not analyzed here. Two defining criteria of this generic number line are that (i) there is an actual *mapping* of each individual number (or numerosity) under consideration (e.g., counting numbers within a certain range) onto a specific location on the line, and that (ii) this mapping defines a uni-dimensional space representing numbers with a *metric* (at least approximative) — a distance function. In numerical contexts a standard definition of mapping is “any prescribed way of assigning to each object in one set a particular object in another (or the same) set. Mapping applies to any set: a collection of objects, such as all whole numbers, all the points on a line, or all those inside a circle” [21]. That the mapping defines a space with a metric, means in this case, that it is a one-dimensional space (i.e., a line) representing numbers with a *translation invariant Euclidean metric*: the length of the segment spatially representing the difference of two numbers satisfies the properties of a Euclidean *distance function* in a one-dimensional space, which is invariant under addition (if the mapping is logarithmic, these properties apply on a log-transformed space). The number line, whether linear or logarithmic, thus has essential mapping properties that go well beyond the numerosity-space associations reported for pre-school children and infants [9], [10]. Such associations have been interpreted as evidence of a number line “mapping”, yet they do not assign to each number (or numerosity) in the collection under consideration a particular location in space (criterion i), and thus do not constitute actual number-to-space mappings, let alone define a space with a metric (criterion ii). Indeed, the reported number-space associations in preschool children [9] were obtained on the basis of a bisection task in which participants were asked to indicate the midpoint of a line segment flanked by the numbers (or numerosities) “2” and “9”. A number-space association was said to exist if the mean reported midpoint exhibited a slight bias towards the number (or numerosity) “9”. The reported associations for infants [10] were obtained by means of a looking habituation paradigm. A number-space association was said to exist if infants transferred the discrimination of ordered series of numerosities to the discrimination of an ordered series of line segments. Despite the use of number-space “mapping” in the titles of these reports, no actual number (or numerosity)-to-line mappings were investigated therein. The reported associations do not conform to standard definitions of mapping [21].

In contrast, cases of finger [22] and body-part counting [8] do constitute a basic form of number-to-space mapping (criterion i), which, even exhibit — at least locally — properties of order (e.g., local transitivity and symmetry properties within the 1–5 range when representing numbers with fingers in one hand). Despite exhibiting (local) order, however, these mappings lack crucial metric properties (criterion ii). For instance, if the counting is performed with the hands open and palms up, the spatial distance between the tip of the thumb (for, say, “one”) and the tip of the index finger (for “two”) — corresponding to one unit — is significantly greater than the distance between the index and the middle finger (for “three”), which also corresponds to one unit. Such mappings then do not satisfy the properties of a Euclidean distance function. The same occurs with the distance between “five” and “six” (i.e., the spatial distance between the last finger of one hand and the first finger of the other hand), or between “ten” and “eleven” (the spatial distance between the last finger and the first toe), and so on.

Based on these features of the number line, a defining criterion of the *number line task* is that “participants evaluate the size of numbers and place them at spatial distances relative to the endpoints that are proportional to their psychological distances from those endpoints” [23]. An interesting finding to emerge from the number line task is that children place smaller numbers at the left of the segment and greater numbers at the right, allocating more space to small numbers and less to big numbers in a logarithmically compressed manner [19], [20]. The data support the idea that numerical estimation obeys the ubiquitous psychophysical Weber-Fechner Law that subjective sensation increases proportional to the logarithm of the stimulus intensity. With education and mathematical training the mapping patterns starts to shift gradually, between kindergarten and fourth grade, from a consistently logarithmic pattern to a primarily linear one [19], [20]. In a similar task, villagers from an Amazonian indigenous group with limited number lexicon [24]— the Mundurukú — were reported to behave like young Western children, mapping number stimuli logarithmically [6]. These results led to the interpretation that the number line mapping is indeed a universal intuition, which initially is logarithmic but becomes linear with education [6].

The Mundurukú study, however, mentions that some participants tended to use only the endpoints of the line segment [6] — failing to use the full response continuum — producing what the authors called a “bimodal” response [25]. Surprisingly, the report does not provide any analysis of these data. Bimodal responses in fact violate the defining criteria of the number line task mentioned above since they lack metric properties (criterion ii), and, therefore, cannot be interpreted as number line mappings proper. Moreover, when their frequency is considerably high they demand further investigation. In the Mundurukú study 13 experimental runs out of 35 (37%) were classified as bimodal [25] — a very high percentage considering the claim that the intuition of the number line mapping is universally spontaneous. According to this claim no runs should be expected to be bimodal. In fact, even if as many as 20% of the runs were expected to be bimodal, the observed frequency of Mundurukú bimodal responses is still statistically significant (χ^2^ = 6.43, df = 1, p = 0.011), casting doubt on the conclusion that indigenous people without instruction spontaneously operate with number line intuitions.

The spontaneity of the number line intuition in unschooled indigenous groups thus requires further investigation. There is, of course, little question that the number line is *learnable*. Given favorable conditions, the most rudimentary features of the number line may be learnable in a matter of hours or even minutes, depending on individual cognitive profiles, pedagogical strategies, supporting materials, and cultural context. Instead, what is at stake is the interesting open question of whether the number line intuition — as the foremost construal brought forth when presented with a line and asked to map numbers onto it — pre-exists any formal instruction and other cultural practices. To date, no case of a culture lacking the spontaneous number line intuition has been empirically demonstrated. Since number line mappings are, in the modern world, ubiquitously embedded in measuring artifacts such as rulers and timelines, dissociating number and number line concepts is methodologically challenging.

Following previous investigations [6], in the present study we used a number line task to test for spontaneous number line intuitions (as indexed by the foremost mapping construal) in the Yupno, an indigenous group from the remote mountains of Papua New Guinea's Finisterre Range. Like other indigenous groups in Papua New Guinea [8], the Yupno have a body-count system, which establishes a number to space mapping that exhibits (local) properties of order but which lacks a metric — that is, a distance function. (By local order we mean that properties of order [e.g., transitivity and symmetry] are exhibited within certain numerical ranges, such as 1–5 as represented by fingers of one hand, but they are not preserved for the body as a whole. Overall spatial order is disrupted several times, as in the passage from locations denoting, for instance, 10 and 11 where the thumb of second hand [number 10] is not followed by a body location “next” to the thumb, but by a toe at the lower extreme of the body [number 11]. Similar disruptions with other body locations representing numbers occur with higher numbers). The Yupno also have a number lexicon beyond twenty and access to a creole (*Tok Pisin*) with English-based number lexicon, but they lack tools and practices for precise space or time measurements [26]. Other groups in Papua New Guinea have been reported to have some established simple measuring practices, such as using arm extension for measuring the depth of string bags [27] but the Yupno do not exhibit them.

20 Yupno adults (14 unschooled and 6 with middle-school education) with demonstrated exact 1–10 cardinal number understanding were individually tested in a number line mapping task (see *Procedure*). Participants were shown a printed line segment introduced as a “path going from one end to the other.” Number stimuli 1 and 10 were then introduced as anchors corresponding to the left and right end of the segment, respectively, and were used as the only training trails. As in previous studies, “because training did not involve intermediate numbers, performance on all subsequent trials served to reveal whether the participants would spontaneously use systematic mapping” ([6] p. 1217), that is, bring forth a specific mapping construal. Number stimuli were presented symbolically (pre-recorded Yupno words) and nonsymbolically (sets of dots, and sequences of tones) in randomized blocks. Since one of the main goals of the study was to test the genuine spontaneity of number line mappings, task instructions were carefully scripted with specific wordings and gestures intended to avoid any unwitting scaffolding. A first version of the instructions had two number-anchors (1 and 10) and static descriptions (Type-1). A second version — designed to be more explanatory — had three number-anchors (1, 10, and 5) and dynamic descriptions (Type-2). Type-1 instructions followed previous studies [6] as closely as possible, while Type-2 instructions were added in the present study to test whether a richer explanation making explicit use of the extent of the path might spark the number line intuition. Each block started with instructions describing the path and indicating the location of the number endpoint anchors 1 and 10 on the line segment. Following anchoring presentations participants were asked to locate the endpoint anchors — 1 and 10 (randomly presented) — one at a time. These served as training trails. If participants failed to correctly assign the anchor points, the instructions were repeated. If for a given block participants failed to correctly locate the anchor points three consecutive times we considered it an endpoint-matching failure and moved on to the next stimulus modality. After successfully passing the anchoring training trials, participants were told that other stimuli would be presented and that, for each one, they were to indicate where it would go on the path. Trials proceeded without feedback. Pointings were video-recorded from above, and digital screenshots of each pointing were extracted for analysis.

## Materials and Methods

### 1. Ethics Statement

This study was conducted in accordance with the guidelines of the University of California, San Diego, Human Research Protections Program. It is part of a project (#080349S) that was reviewed and approved by one of this institution's Institutional Review Boards in accordance with the requirements of the Code of Federal Regulations on the Protection of Human Subjects (45 CFR 46 and 21 CFR 50 and 56), including its relevant Subparts. Additionally, one of the researchers (JW) has been granted explicit, written permission by members of the Yupno community to conduct fieldwork in the village of Gua and has done so for more than 25 years. Recruitment of participants was facilitated by local, bilingual (Yupno-English) field assistants who were respected members of the community. A few individuals declined to participate. Participants who volunteered, being predominantly illiterate and largely unfamiliar with Western conventions and protocols could not give written consent so they gave it orally. Oral consent, while not explicitly mentioned in the above IRB approval, has been the practice at Gua for many years and is in accordance with the permissions obtained by JW. Consent was documented immediately before experimentation and was witnessed by a Yupno bilingual field assistant who was always present during the task should questions or concerns arise. In the extremely rare cases where the task seemed to be causing distress or embarrassment, the experiment was terminated with no indication that the participant's performance was unsatisfactory. In carrying out this research we conformed to the standards of the Declaration of Helsinki (especially article 24) in its latest version.

### 2. Participants

The study was carried out in the remote Upper Yupno valley of the Finisterre Range, Papua New Guinea, in August-September 2009. The Upper Yupno community, spread over various small villages, has a population of about 5000, and has no electricity or roads. (All research equipment thus had to be powered by portable solar panels and batteries.) The community is largely illiterate, and lives by subsistence farming [28], [29]. Initially, 26 Yupno adults participated in the study (age range approximately 20–65; 20 unschooled (10 women) and 6 with middle-school education (1 woman)). 6 unschooled adults were eliminated from further analysis after failing the cardinal number-lexicon screening. Because the Yupno do not measure age, estimations were obtained separately from two informants. Educational levels among adult Yupno villagers vary substantially, and so does curriculum implementation. We defined the relevant participant groups for the study as follows: “adult” as 20 or older; “unschooled” as someone who had not been in formal school at any time in the 15 years leading up to testing, and who never advanced beyond 6^th^ grade (17 participants never attended school); and “schooled” as someone who within 10 years prior to testing had attended at least 8^th^ grade. Among the Yupno, education beyond 8^th^ grade is rare, requiring moving to a distant city outside of the valley. 3 participants reached 10^th^ grade, a rare accomplishment. 1 was a woman, tested in the city of Madang. Controls were 10 adults from San Diego, California (age range 20–64; 4 women). Their responses fully replicated previous studies [6].

### 3. Procedure

#### 3.1. Cardinal number-lexicon screening

To make sure that unschooled Yupnos participating in the number line task understood 1–10 cardinal numerical lexicon we established a simple screening procedure. Participants, tested individually, heard a pre-recorded voice randomly utter numbers from 1 to 10 in the Yupno language. For each spoken number stimulus they were asked to pick the corresponding number of items from a pile of fruits (see Fig. S1). Participants were considered to have failed the number screening if they made more than one error. This was the case of six unschooled Yupno participants, who did not proceed to the next stage with the number line task. To cover dialectal variation, two equivalent recordings were employed. Participants' preferred version was used. The percentage of success in the number-lexicon screening for the 14 selected unschooled Yupno participants was 96.4%, and for all 6 schooled ones was 100%.

#### 3.2. Number line task

Participants were presented a black 22 cm-long line segment printed on a white 21.5×28 cm card placed in front of them on the floor. The segment was visually similar to one used previously with an isolated non-Western culture [6], but, to increase ecological validity, it was displayed on paper (not a computer screen), and required participants to sit on the floor as is customary. Moreover, the line did not have any other accompanying depiction (e.g., 1 dot and 10 dots at the left and right endpoints, respectively) in order to avoid possible confounds involving perceptual resemblance that could differentially influence responses to the various stimulus modalities (see Fig. S2). More specifically, depicting 1 and 10 dots at the line's endpoints may influence responses to number stimuli presented as dots — which are perceptually similar to such a depiction — differently than those presented as words or tones — which are qualitatively different from the depiction. Number stimuli were presented in three modalities: visually as sets of black dots printed on white 21.5×28 cm cards, auditorily as sequences of tones, and as pre-recorded spoken words. Each individual testing consisted of three randomized blocks, one per stimulus modality, which in turn included two randomized runs through the 1–10 number set (20 trials per block). Each block started with the presentation of the line segment (see Fig. S3 for wording details), followed by the introduction of the number stimuli 1 and 10 as anchors corresponding to the left and right end of the segment, respectively. As training trials, we then randomly presented either number stimulus 1 or 10 and asked the participant to point to its corresponding location on the path. These number stimuli were the only training trails used.

Because one of the main goals of the study was to investigate the genuine spontaneity of number line intuitions as indexed by the foremost mapping construal we carefully scripted the instructions with specific wordings and gestures. We did not deviate from the script and did not provide further elaboration. Fig. S3 shows a description of the instructions as well as their morpheme-by-morpheme gloss. 8 unschooled Yupnos received Type-1 instructions, and 6 unschooled Yupnos received Type-2 instructions. All 6 schooled Yupnos and adult control participants from the San Diego area received Type-1 instructions.

Pointings were video-recorded from the top (approximately 1.6 m from the floor) with a Sony DCR-PC110 digital camera (see Fig. S2 for examples). On average screenshots rendered the segment as a 656-pixel-long object. Response locations were measured by the distance (in pixels) between the segment's left endpoint and the middle of the pointing finger and transformed to a 1–10 scale.

#### 3.3. Notes on statistical analysis

Responses in 6 blocks of 1–10 number sets (3 from two unschooled Yupnos, and 3 from two schooled ones) had a non-significant intra-subject correlation between the two trials, thus lacking validity. These blocks were excluded from the reported regression analyses.

Ten sections were defined for the analysis of proportions of responses on the segment reported below. Sections for the eight intermediate numbers *k* ( = 2, 3, …, 9), included responses falling in the interval [*k*−0.5, *k*+0.5). The critical left and right endpoint intervals only included responses that were smaller than 0.5 and larger than or equal to 9.5, respectively.

## Results

**Figure 1:**
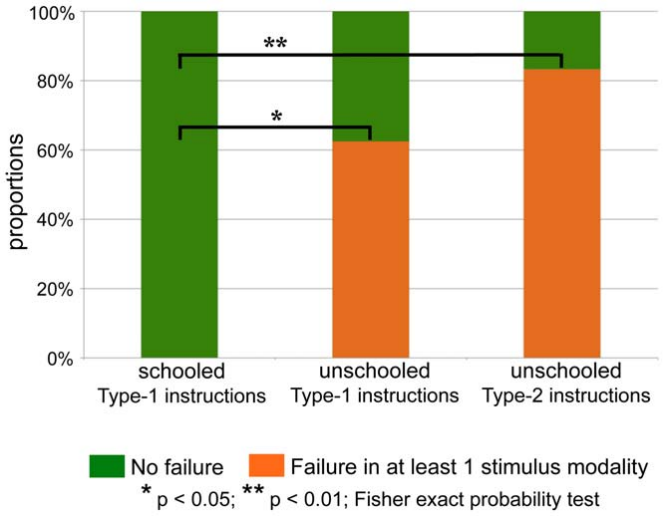
Results of endpoint matching during the training trials. Proportions of failures in the training trials 1 and 10 (endpoint anchoring), after succeeding at a number-lexicon screening. Despite succeeding in the number screening, unschooled Yupnos exhibited a significantly higher proportion of failure when matching endpoint anchors than schooled Yupnos. The more explicit Type-2 instructions did not help.

Unlike schooled Yupnos, and despite having passed the cardinal number understanding screening, unschooled participants had serious difficulties with the training trails, failing to understand the fundamental endpoint anchoring required by the number line task (Fig. 1). 5 out of 8 (62.5%) receiving Type-1 instructions and 5 out of 6 (83.3%) receiving Type-2 instructions, failed to match — in at least one stimulus modality — the endpoint anchors 1 and 10 (3 times) during the training trails. These proportions of failures are significantly higher than those observed for the schooled Yupno participants (all of whom passed the number screening), who exhibited no endpoint-matching failures (0 out of 6, 0%) (Fisher exact probability test, one-tailed p = 0.028 and p = 0.008, for unschooled Yupnos receiving Type-1 and Type-2 instructions, respectively). Most importantly, the Type-2 instructions, which involved dynamic language and which explicitly showed the mapping of the number 5 onto a location on the line between the anchors 1 and 10, did not help.

Crucially, analyses performed with blocks that had successful endpoint anchoring trials under Type-1 instructions show that unschooled Yupnos consistently ignored the extent of the path, producing — in all three stimulus modalities — a metric-free bi-categorical mapping, where small numbers (1, and sometimes 2 and 3) mapped onto the left-endpoint and the rest onto the right-endpoint (see Table 1 for details). The median response location for number stimulus 1 was 1.07 (indicating left-endpoint location) and each of the median response locations for number stimuli larger than 2 was essentially 10 (thus indicating right-endpoint location). The median for number 2 was 9.82, reflecting some variability in the choices between left- and right-endpoint with a strong bias towards the right-endpoint. The interquartile ranges show that the number stimulus that had by far the highest variability was number stimulus 2 (8.88), followed by number stimulus 3 (2.63). All other number stimuli had inter-quartile ranges smaller than 1.0, reflecting a high degree of homogeneity in the response location on the endpoint — left-endpoint for number stimulus 1, and right-endpoint for all number stimuli greater than 3. These data show that for the construed bi-categorical mapping the shifting numerical magnitude between the two categories is around the number 2.

**Table 1:** Unschooled Yupno response locations on number line task.

Number stimulus	Median response location	25^th^ percentile	75^th^ percentile	Interquartile range
**1**	1.07	0.99	1.17	0.18
**2**	9.82	1.15	10.03	8.88
**3**	10.01	7.47	10.10	2.63
**4**	10.01	9.28	10.09	0.81
**5**	9.99	9.90	10.07	0.17
**6**	9.99	9.91	10.04	0.13
**7**	9.99	9.84	10.05	0.21
**8**	9.99	9.94	10.13	0.19
**9**	10.01	9.88	10.06	0.18
**10**	10.01	9.97	10.03	0.06

Number line task response locations by unschooled Yupno participants receiving Type-1 instructions (all blocks combined).

Interestingly, the sole unschooled Yupno participant receiving Type-2 instructions (with 3 anchors corresponding to 1, 10, and 5) who exhibited no endpoint matching failures during the training trails, also systematically mapped intermediate numbers in all blocks onto categories. But this participant mapped number stimuli categorically onto three locations: small numbers 1–2 on the left-endpoint, large numbers 6–10 on the right-endpoint, and mid-size numbers 3–5 at the center (with minor range variations depending on stimulus modality). The resulting tri-categorical mapping, ignoring the extent of the path, is metric-free, and illustrates the primacy of categorical thinking in the Yupno mind.

**Figure 2:**
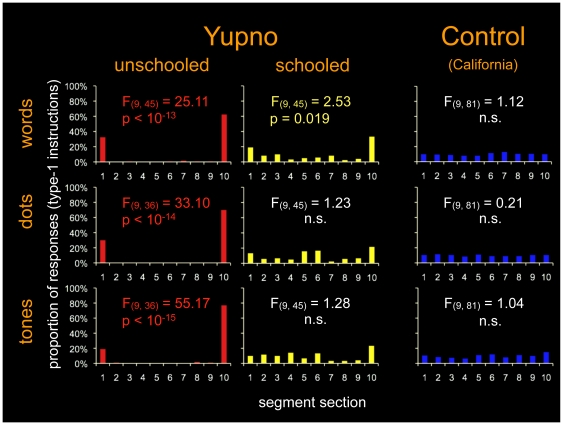
Responses on the number line task. Proportions of responses on segment sections with repeated measures ANOVA statistics. In sharp contrast with schooled Yupnos (yellow) and California controls (blue), unschooled Yupnos (red) did not employ the extent of the segment when mapping intermediate numbers, exhibiting a bi-categorical mapping without a metric (a distance function). Schooled Yupnos (yellow) did employ the line, but exhibited a bias towards the endpoints in at least one stimulus modality (words).

The bi-categorical responses exhibited by unschooled Yupnos receiving Type-1 instructions, which did not employ the extent of the segment when mapping intermediate numbers, are in sharp contrast with those of schooled Yupnos and California controls (Fig. 2). Repeated measures ANOVAs show that the high proportions of endpoint responses by unschooled Yupno were extremely significant in all three stimulus modalities (words: F(9, 45) = 25.11, p = 1.01×10^−13^ ; dots F(9, 36) = 33.10, p = 8.49×10^−15^; tones F(9, 36) = 55.17, p = 2.2×10^−16^). California controls systematically used all sections of the segment in all three stimulus modalities (words: F(9, 81) = 1.12, p = 0.360; dots F(9, 81) = 0.21, p = 0.993; tones F(9, 81) = 1.04, p = 0.415). Furthermore, while schooled Yupnos did employ the line, they exhibited a clear bias towards the endpoints in at least one stimulus modality (words: F(9, 45), 2.53, p = 0.019; dots F(9, 45) = 1.23, p = 0.301; tones F(9, 45) = 1.28, p = 0.274).

**Figure 3:**
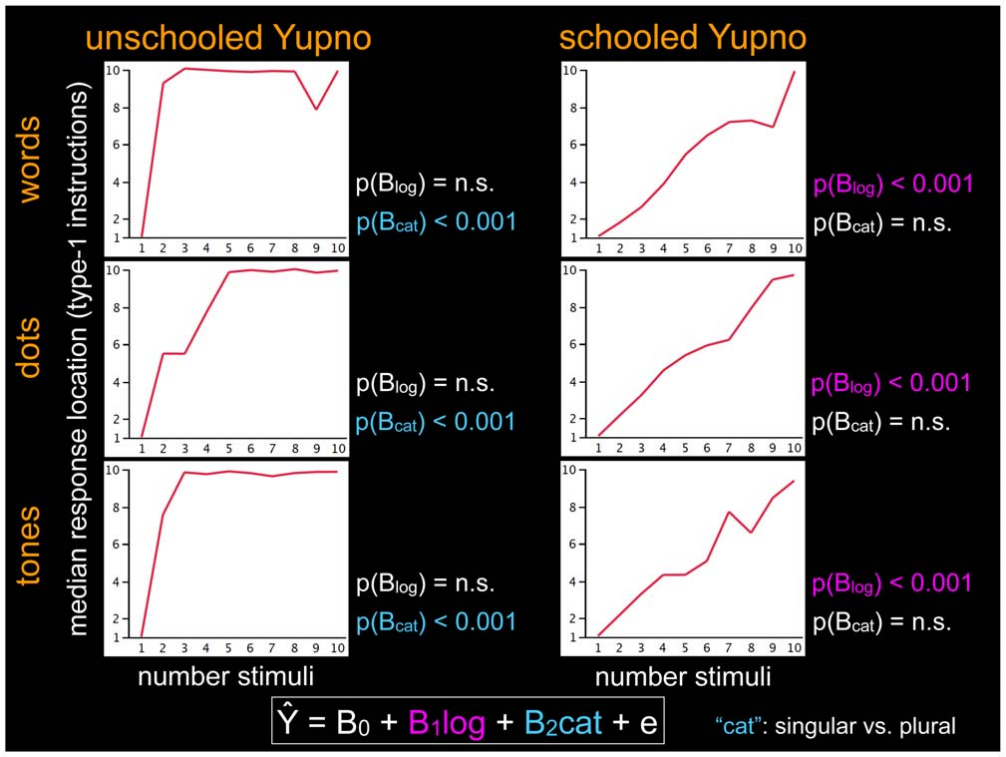
Median regression models of Yupno responses on number line task. Median response locations on the segment by unschooled and schooled Yupnos, with corresponding p-values for regressors' weight in median regressions with logarithmic and bi-categorical predictors. The results show a clear dissociation of the predictors needed to model the responses of the two Yupno groups. For the unschooled Yupno the bi-categorical regressor (light blue) — but not the logarithmic regressor (purple) — is significant for all three stimulus modalities, while the opposite is true for the schooled Yupno.

We further analyzed the response locations among the unschooled Yupno through median regressions with logarithmic [6], [20] and singular-plural bi-categorical regressors. The weights of the bi-categorical regressors (B_cat_) — but not the logarithmic ones (B_log_) — were consistently significant for all three stimulus modalities in unschooled Yupnos, while exactly the opposite pattern was observed in schooled Yupnos (Fig. 3; unschooled Yupno: p(B_cat_)<0.001 and p(B_log_) is not significant in all three stimulus modalities; schooled Yupno: p(B_cat_) is not significant and p(B_log_)<0.001 in all three stimulus modalities). These results provide a clear dissociation of the predictors needed to model the responses of schooled and unschooled Yupnos, suggesting a fundamental difference in the nature of the mappings produced by these two Yupno groups: the former exhibit a mapping with a standard nonlinear compression [19], [6], [20] while the latter produce instead a metric-free bi-categorical mapping.

**Figure 4:**
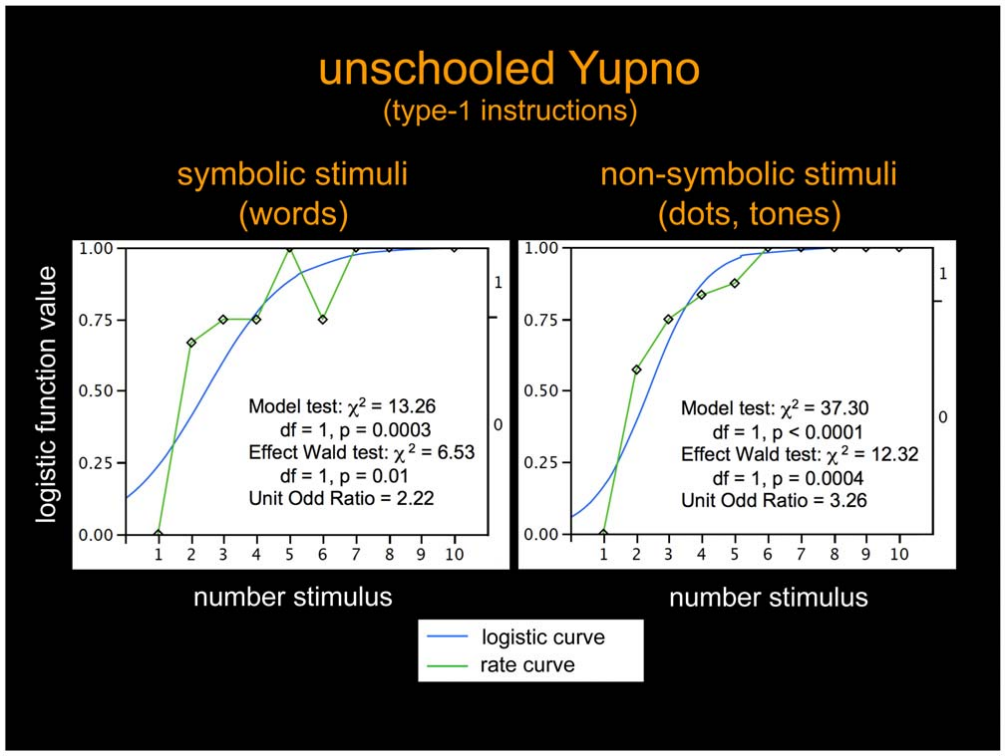
Logistic regression models of unschooled Yupno responses. Logistic regressions successfully modeling unschooled Yupnos' bi-categorical responses for both, symbolic and non-symbolic stimuli, with corresponding rate curves (green) and logistic curves (blue). These regressions provide a valid model for response locations to intermediate number stimuli in terms of likelihoods of being placed at one end or the other of the segment.

It is tempting to interpret response location curves based on central tendency statistics as corresponding to actual locations on the line segment [6], for instance, by reading all median values in Fig. 3 as characterizing proper locations on the line segment. From the data illustrated in Fig. 2, however, we see that while this reading is appropriate for the schooled Yupno, it is evidently not so for the unschooled Yupno, since the latter group did not produce responses located along the extension of the line segment. Consequently, we analyzed the nonlinearity of the unschooled Yupno curve (Fig. 3 left column) with logistic regressions rather than with regressions containing standard linear and logarithmic regressors [6], [20]. Logistic regressions on the endpoint bi-categorical unschooled Yupno responses successfully modeled the data (Fig. 4). The model test was highly significant for both, symbolic (χ^2^ = 13.26, df = 1, p = 0.0003) and non-symbolic (χ^2^ = 37.30, df = 1, p<0.0001) stimulus modalities (Effect Wald test: χ^2^ = 6.53, df = 1, p = 0.01 for symbolic stimuli, and χ^2^ = 12.32, df = 1, p = 0.0004 for non-symbolic stimuli; Unit Odd Ratio: 2.22 for symbolic stimuli and 3.26 for non-symbolic stimuli). These logistic regressions provide a valid model for bi-categorical response locations to intermediate numbers in terms of likelihoods of being placed at one end or the other of the segment, rather than as proper locations on the segment.

## Discussion

Our results show that adults from the isolated and largely unschooled Yupno community in the remote mountains of Papua New Guinea, despite having precise cardinal number concepts, do not spontaneously exhibit number line intuitions when presented with an external line. First, unlike schooled Yupnos, unschooled participants had serious difficulties understanding the fundamental endpoint anchoring required by the number line task. Importantly, more explicit instructions showing the mapping of the intermediate number stimulus 5 onto a location between the anchors 1 and 10 did not help. Second, in the cases where unschooled Yupno participants did establish the endpoint anchors in the training trials, the resulting mapping exhibited a bi-categorical pattern with intermediate values mapped onto the segment's endpoints, thus violating fundamental metric properties of the number line — the crucial criterion ii described above.

These Yupno bi-categorical responses seem to be of the same nature as the frequent “bimodal” responses — mentioned but left unanalyzed — in the Amazonian study among the Mundurukú [6]. In both cases, the modes of the distributions are concentrated at the endpoints, and, in both cases, the volume of responses failing to use the extension of the line segment to map intermediate number stimuli is significant. This suggests that the bi-modal Mundurukú responses are not simply anomalies that can be ignored, but are in fact clear-cut evidence of a real phenomenon of lack of spontaneous number line intuitions. Mundurukú endpoint responses, although significant, were not as pronounced as they were among the unschooled Yupno. A possible explanation is that Mundurukú participants were presented with a line that — at all times and for all conditions — had a depiction of 1 dot and 10 dots located at the left and right end of the segment, respectively. This may have provided extra help to participants in the form of a perceptual resemblance cue, which would explain why, for number stimuli “one”, “two”, and “three” (the only terms Mundurukú participants match to collections of corresponding quantities in more than 70% of the cases [30]), unschooled adult Mundurukú participants provided average response locations preserving order for dot number stimuli but not for non-visual stimuli (words and tones) [25]. Taken together, the results from both the present study and the earlier Amazonian study suggest that the number line intuition does not manifest universally in a spontaneous manner.

Finally, schooled Yupnos used the extent of the segment according to known standards [6], [20]. Interestingly, however, they did so not as evenly as western controls, exhibiting a bias towards the endpoints. The schooled Yupno response pattern suggests a culture-driven intermediate facility with the number line, in which elements of the basic bi-categorical intuition co-exist with the learned metric of the number line mapping. These findings demonstrate that the intuition of the number line mapping as reflected by the number line task is not universally spontaneous, and therefore, unlikely to be rooted directly in brain evolution. Such a conclusion is consistent with recent findings showing that number-to-space mappings are not as fundamental as previously thought, and that linear number line mappings require cultural practices to be established [31]. Moreover, these observations also seem to match the available records of the history of mathematics, which show no documentation of depictions of number lines proper prior to the 17^th^ Century [4], [32]. The number line seems to explicitly appear for the first time with the work of John Napier [33] and John Wallis [34] as a response to demands imposed by the conceptualization of more sophisticated mathematical objects, such as logarithms (Napier) and negative squares and their imaginary roots (Wallis). In some well-known cases, cultures appear to have developed sophisticated forms of arithmetic, and mathematics in general, without number lines. In Babylonia, for instance, out of the roughly 500,000 known tablets, only about 50 of them contain mathematical depictions and not a single one depicts or discusses number lines proper [35]. The number-to-line mapping does not seem to have been part of the Babylonian repertoire of mathematical cognitive techniques. Until very late indeed (3^rd^ century BC) number was conceptualized essentially as an *adjectival property* of a collection or of a measured object [35], which suggests that the use of measurements does not necessarily imply the concept of number line proper.

In sum, evidence from a variety of sources — including developmental work on infants and cross-cultural work on counting practices — points to a basic human association between number and space. But the present results suggest that the emergence of the *number line* proper, in which numbers are mapped to unidimensional space with a metric (with a distance function), requires additional cultural factors and scaffolding. Specific cultural practices, such as measurement tools, graphical representations, writing systems, and systematic education, serve to introduce, refine, and continually reinforce the particulars of the number line mapping. It is likely over the course of extended exposure to these cultural practices that brain areas such as the parietal lobes are recruited to support number representation and processing [36].

## Supporting Information

Figure S1 **Number lexicon screening task.** Participant matching quantity to auditorily presented Yupno number words during the cardinal number-lexicon screening.
(TIF)

Figure S2 **Example responses on number line task.** Pointing examples for stimulus numbers 3 and 6 (dots) during the number line task.
(TIF)

Figure S3 **Instructions for number line task.** Description of the instructions for the number line task, including a morpheme-by-morpheme gloss of the Yupno expressions employed.
(TIF)
