# Correction: Number Concepts without Number Lines in an Indigenous Group of Papua New Guinea

**DOI:** 10.1371/annotation/f2b5bdcf-d215-4617-bd34-f0194809df73

**Published:** 2013-10-15

**Authors:** Rafael Núñez, Kensy Cooperrider, Jürg Wassmann

Supplementary Figures 1 and 2 were incorrectly switched. The image currently appearing as Supplementary Figure 1 belongs with the title and legend for Supplementary Figure 2, and the image currently appearing as Supplementary Figure 2 belongs with the title and legend for Supplementary Figure 1. The titles and legends of the figures are in correct order. 

