# Supplementary figures and images for: Number Concepts without Number Lines in an Indigenous Group of Papua New Guinea
Source: PLoS One. 2012 Apr 25;7(4):e35662. doi: 10.1371/journal.pone.0035662 (PMC3338449; doi:10.1371/journal.pone.0035662)

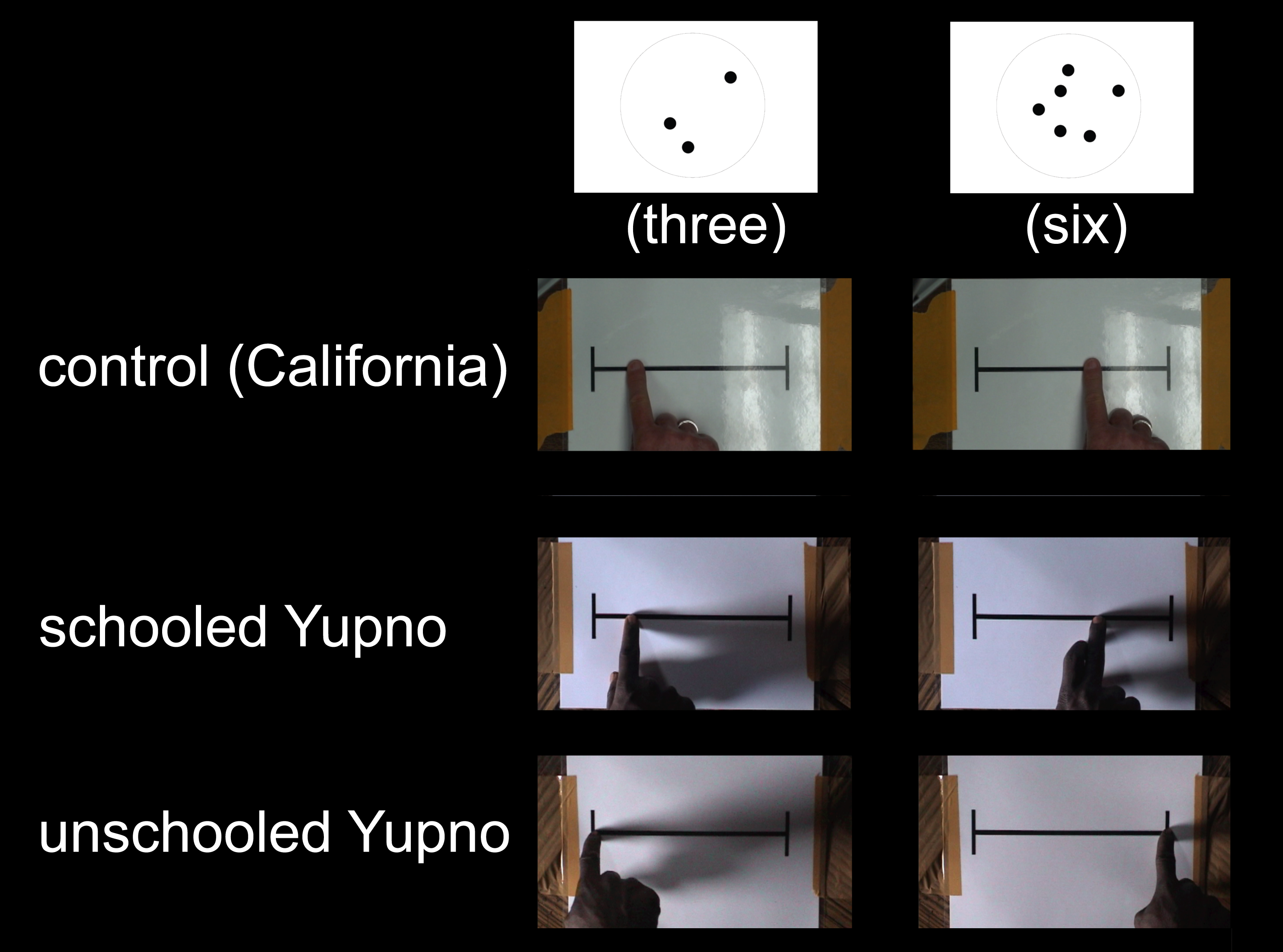

Supplement: Figure S1 — Number lexicon screening task. Participant matching quantity to auditorily presented Yupno number words during the cardinal number-lexicon screening. (TIF) [file pone.0035662.s001.tif]

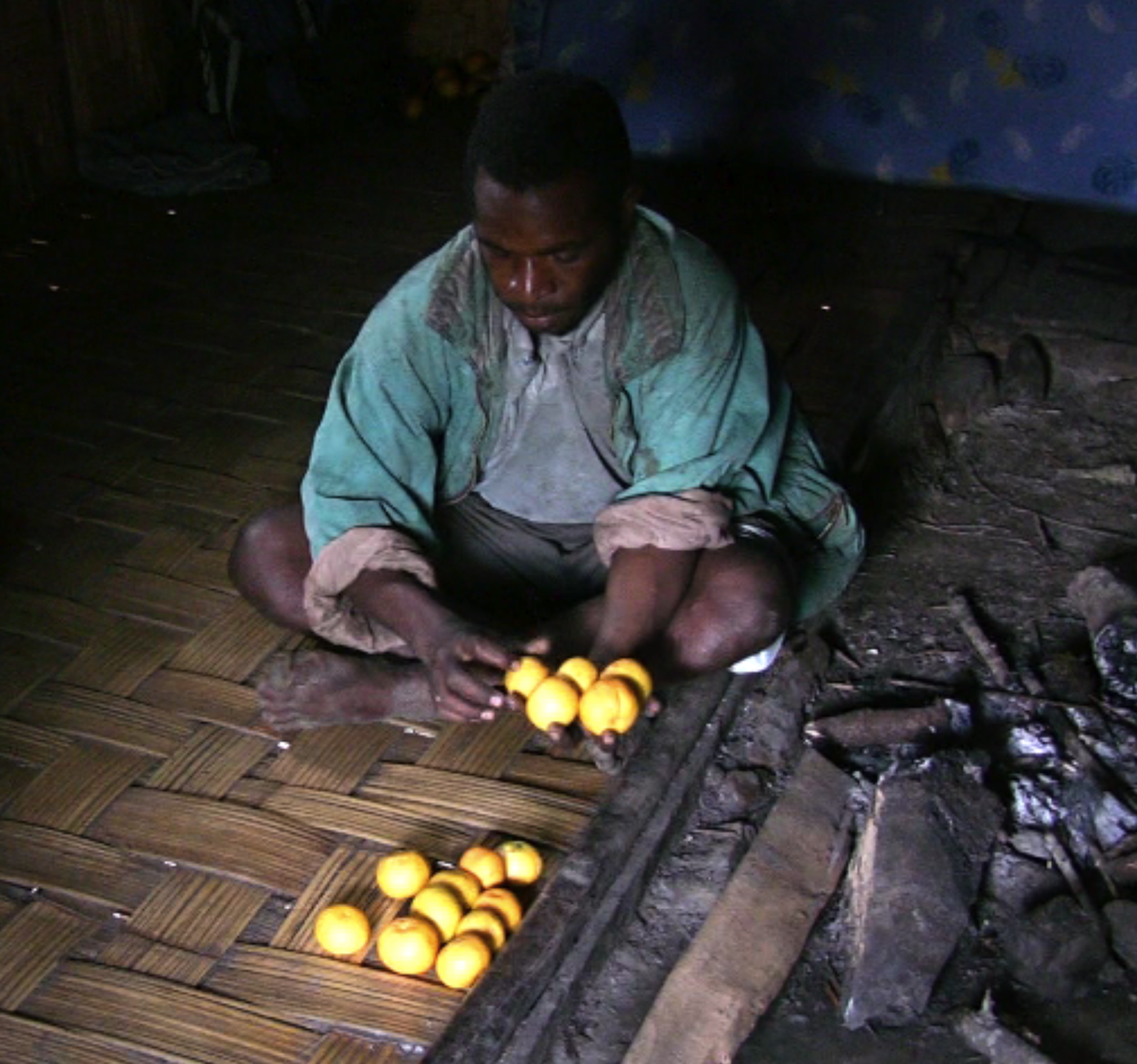

Supplement: Figure S2 — Example responses on number line task. Pointing examples for stimulus numbers 3 and 6 (dots) during the number line task. (TIF) [file pone.0035662.s002.tif]

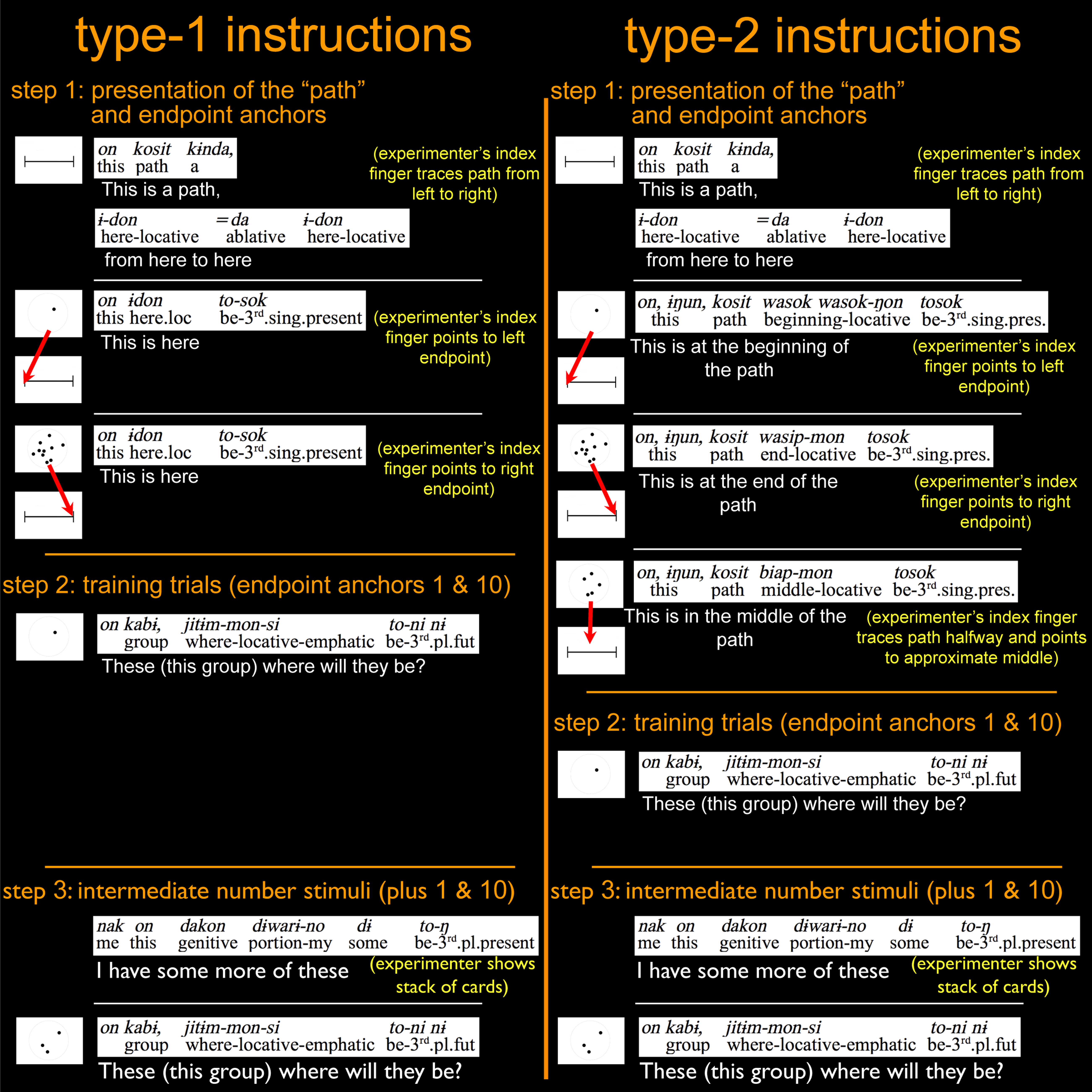

Supplement: Figure S3 — Instructions for number line task. Description of the instructions for the number line task, including a morpheme-by-morpheme gloss of the Yupno expressions employed. (TIF) [file pone.0035662.s003.tif]
